# Cell Origin and iNOS Function Are Critical to Macrophage Activation Following Acute Lung Injury

**DOI:** 10.3389/fphar.2021.761496

**Published:** 2022-01-25

**Authors:** Thea N. Golden, Alessandro Venosa, Andrew J Gow

**Affiliations:** ^1^ Center for Research on Reproduction and Women’s Health, School of Medicine, University of Pennsylvania, Philadelphia, PA, United States; ^2^ Center for Excellence in Environmental Toxicology, School of Medicine, University of Pennsylvania, Philadelphia, PA, United States; ^3^ Department of Pharmacology and Toxicology, University of Utah, Salt Lake City, UT, United States; ^4^ Department of Pharmacology and Toxicology, Rutgers, The State University of New Jersey, Piscataway, NJ, United States

**Keywords:** pulmonary injury, pulmonary inflammation, iNOS inhibition, macrophage activation, macrophage phenotype

## Abstract

In the intratracheal bleomycin (ITB) model of acute lung injury (ALI), macrophages are recruited to the lung and participate in the inflammation and resolution that follows injury. Macrophage origin is influential in determining activation; however, the specific phenotype of recruited and resident macrophages is not known. Inducible nitric oxide synthase (iNOS) has been implicated in the pathogenesis of ALI; however, the effects of its inhibition are mixed. Here we examined how macrophage origin determines the phenotypic response to ALI. Further, we hypothesize cell specific iNOS is key to determining activation and recruitment. Using a chimeric mouse approach, we have identified recruited and resident macrophage populations. We also used the chimeric mouse approach to create either pulmonary or bone marrow NOS2^−/−^ mice and systemically inhibited iNOS via 1400 W. We evaluated macrophage populations at the peak of inflammation (8 days) and the beginning of resolution (15 days) following ITB. These studies demonstrate tissue resident macrophages adopt a M2 phenotype specifically, but monocyte originated macrophages activate along a spectrum. Additionally, we demonstrated that monocyte originating macrophage derived iNOS is responsible for recruitment to the lung during the inflammatory phase. Further, we show that macrophage activation is dependent upon cellular origin. Finally, these studies suggest pulmonary derived iNOS is detrimental to the lung following ITB. In conclusion, macrophage origin is a key determinant in response to ALI and iNOS is central to recruitment and activation.

## Introduction

Acute lung injury (ALI) is a multi-stage process that initiates with an insult to the lung and progresses through inflammatory and resolution phases. Excess inflammation early in the process results in increased acute injury, while overly aggressive resolution produces fibrosis. The initiating agent is a critical determinant of the time course and progression of the injury process. Activated bleomycin can produce oxidative damage to DNA (Burger et al., 1981) and so result in epithelial injury following instillation into the lung. The use of intratracheal instillation of bleomycin (ITB) has become a widely used model to study the stages of ALI following epithelial injury, namely inflammation, resolution, and fibrosis (Liu et al., 2017). The time course of these phases is relatively well-established with inflammation dominating in the first week after injury followed by a resolution phase that can last up to 2 weeks (Savani et al., 2001; Walters and Kleeberger, 2008).

Macrophages are the key players in the response to ALI (Cavarra et al., 2004; Ishida et al., 2017), whose activation along a phenotypic spectrum is dependent both upon the stimulus and the origin of the cells (Okuma et al., 2004; Trujillo et al., 2008; Martinez and Gordon, 2014; Arora et al., 2018). Macrophages are abundant during the acute inflammatory stage following ITB and are classically activated (Cavarra et al., 2004; Gasse et al., 2007). As inflammation resolves, macrophages favor alternative activation (Cavarra et al., 2004; Gasse et al., 2007). Macrophages during ALI are either resident to the lung (Tissue Resident Macrophages, TRM), therefore present at the time of injury, or recruited to the lung in response to injury (Monocyte-derived Macrophages, MoM) (Watanabe et al., 2019). Resident macrophages have a particular phenotype to allow for their homeostatic role of interfacing with the environment (Watanabe et al., 2019). Resident alveolar macrophages are highly phagocytic and tolerant (Toews et al., 1984; Guth et al., 2009; Zaynagetdinov et al., 2013). Epithelial cell derived proteins, such as GM-CSF and TGFβ, influence resident alveolar macrophage phenotype (Roth and Golub, 1993; Guth et al., 2009). Recruited macrophages enter the lung via chemokine signaling, thus influencing their phenotype. In the LPS model of ALI, cell origin influenced macrophage transcript (Mould et al., 2017), but the signaling mechanisms that govern origin-related macrophage activation are unknown.

Nitric oxide is a potent signaling molecule that can be produced by multiple cell types including macrophages themselves (Lancaster and Hibbs, 1990). It has been shown to influence macrophage phenotype by regulating intracellular metabolism (Bailey et al., 2019). Previously, we have shown that iNOS inhibition within the ITB model leads to a reduced inflammatory response; however, at later time points there is an exacerbation of fibrosis (Guo et al., 2016). Others have also demonstrated impaired resolution in the LPS model of ALI when iNOS is inhibited (D’alessio et al., 2012). This suggests iNOS may play a paradoxical role in that it is detrimental during inflammation but necessary for resolution of inflammation.

In this study, we have examined the effect of ITB on TRMs within the lung and the activation and recruitment of MoMs. Further we have examined how the cellular location of NO generation may be critical in determining the balance between recruited and resident macrophages. We have taken advantage of a chimeric mouse model in which the bone marrow derived cells can be of different genetic origin than the host (Tanaka et al., 2003). It is our hypothesis that both loss of TRMs and subsequent recruitment of MoMs is required for ITB-induced inflammation and that iNOS is an important regulator of macrophage polarization both in resident and recruited cells.

## Materials and Methods

### Study Design

Mice were instilled with bleomycin on day 0 and then analyzed at either 8 or 15 days post instillation in order to examine inflammatory activation at its peak (8 days) or at the initiation of resolution (15 days). Based on our previous experience with the intratracheal bleomycin model of acute lung injury, we collected samples from 3–7 mice at both time points. All endpoints were measured in samples from each mouse. Over the course of the study, mice were all treated with the same lot of bleomycin by the same administrator to ensure similar injury between mice. Outliers were defined as greater than 2 standard deviations from the mean; however, no outliers were identified.

The purpose of this study was to assess the role of macrophages in inflammation and resolution following ITB. The primary objective was to phenotype macrophage populations based on origin and activation state. Our pre-specified hypotheses were (1) origin is a major determinant of macrophage activation following ITB and (2) cell specific iNOS function was influential to recruitment and activation. C57BL/6 mice were used in this study as they are commonly used by us and others in the ITB model. Also, there were both GFP expressing and *NOS2* ^−/−^ strains available on the C57BL/6 background. Mice were randomly chosen for either PBS or bleomycin treatment and cages included both treatment groups so as to limit a cage effect. Mice were assigned a number so as to blind collection, measurement, and analysis.

### Animals

**FIGURE 1:**
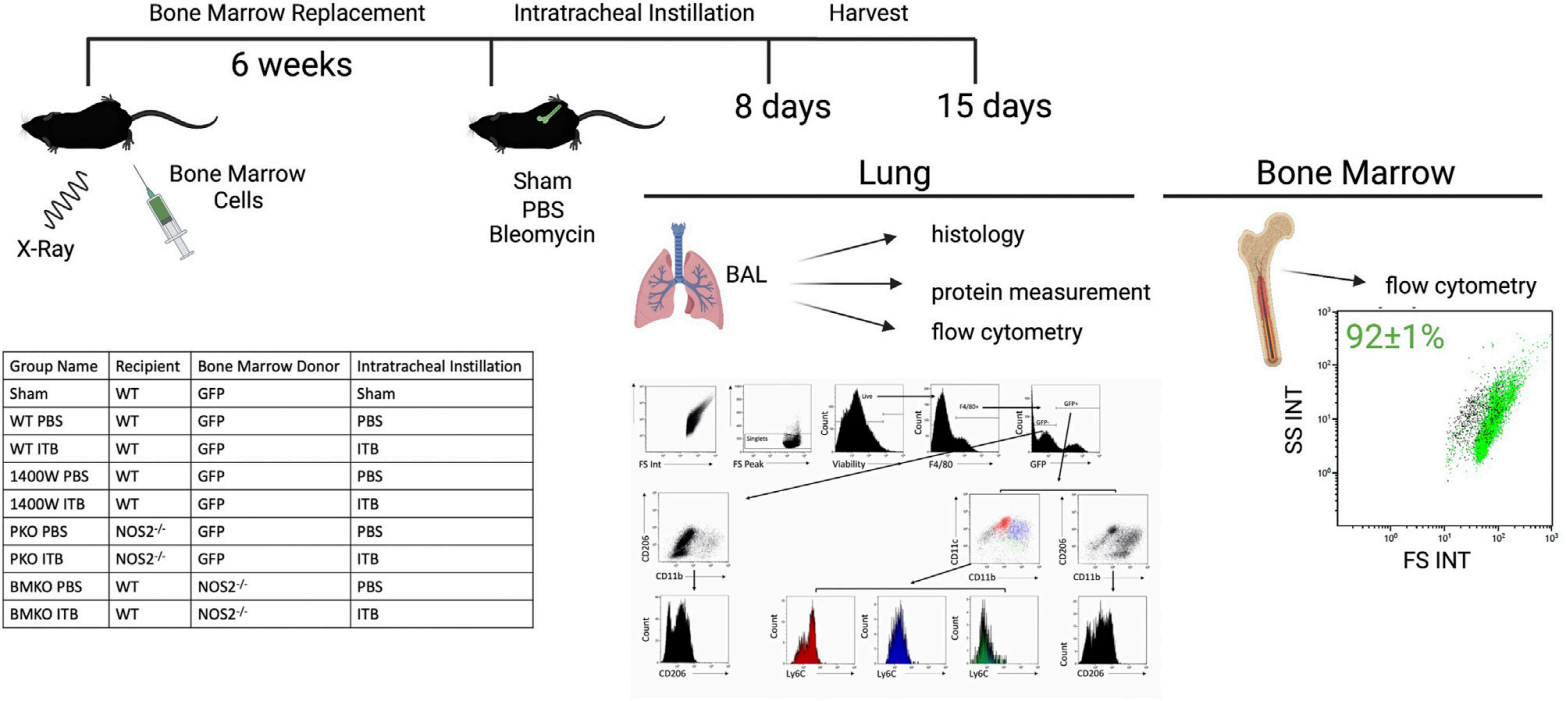
The experimental approach is illustrated to include the method of developing chimeric mice, intratracheal instillation, and endpoints. The table lists the experimental groups. The flow gating strategy employed evaluates live single macrophage (F4/80+) cell expression of GFP, CD11b, CD11c, CD206, and Ly6C in order to assess origin, maturity, and activation collected via BAL. Protein levels were measured in the cell free BAL. Lungs were subsequently histologically evaluated. The percent of GFP+ cells in the bone marrow was measured by flow cytometric detection of GFP expression and reported as mean ± SE. Created with Biorender.com.

Six-week-old male C57/BL6 (Jackson) underwent 12 Grey X-ray radiation using a Torrex irradiator (Figure 1). Twelve Grey X-ray radiation required 45 min. Bone marrow was harvested from C57BL/6-Tg (CAG-EGFP)10sb/J or *NOS2* ^*−/−*^ mice and 1 × 10^6^ cells injected 2–4 h following radiation. C57BL/6-Tg (CAG-EGFP)10sb/J bone marrow reconstituted C57/BL6 mice to create green fluorescent protein (GFP) chimeras. Pulmonary knockout chimeras were generated by replacing the bone marrow of *NOS2* ^*−/−*^ mice with C57/BL6 and bone marrow knockout chimeras by replacing C57/BL6 with *NOS2* ^*−/−*^ bone marrow. Mice were monitored for 6 weeks and fed PicoLab 0.025% Trimethoprim/0.1242% Sulfamethoxazole antibiotic chow for the first 2 weeks (W.F. Fisher and Son Sommerville, NJ).

GFP expression in bone marrow cells was measured to ensure reconstitution. Bone marrow cells were gated on forward and side scatter to exclude red blood cells (GFP-). A total of 92 ± 1% of bone marrow cells were GFP+ (Figure 1).

**FIGURE 2:**
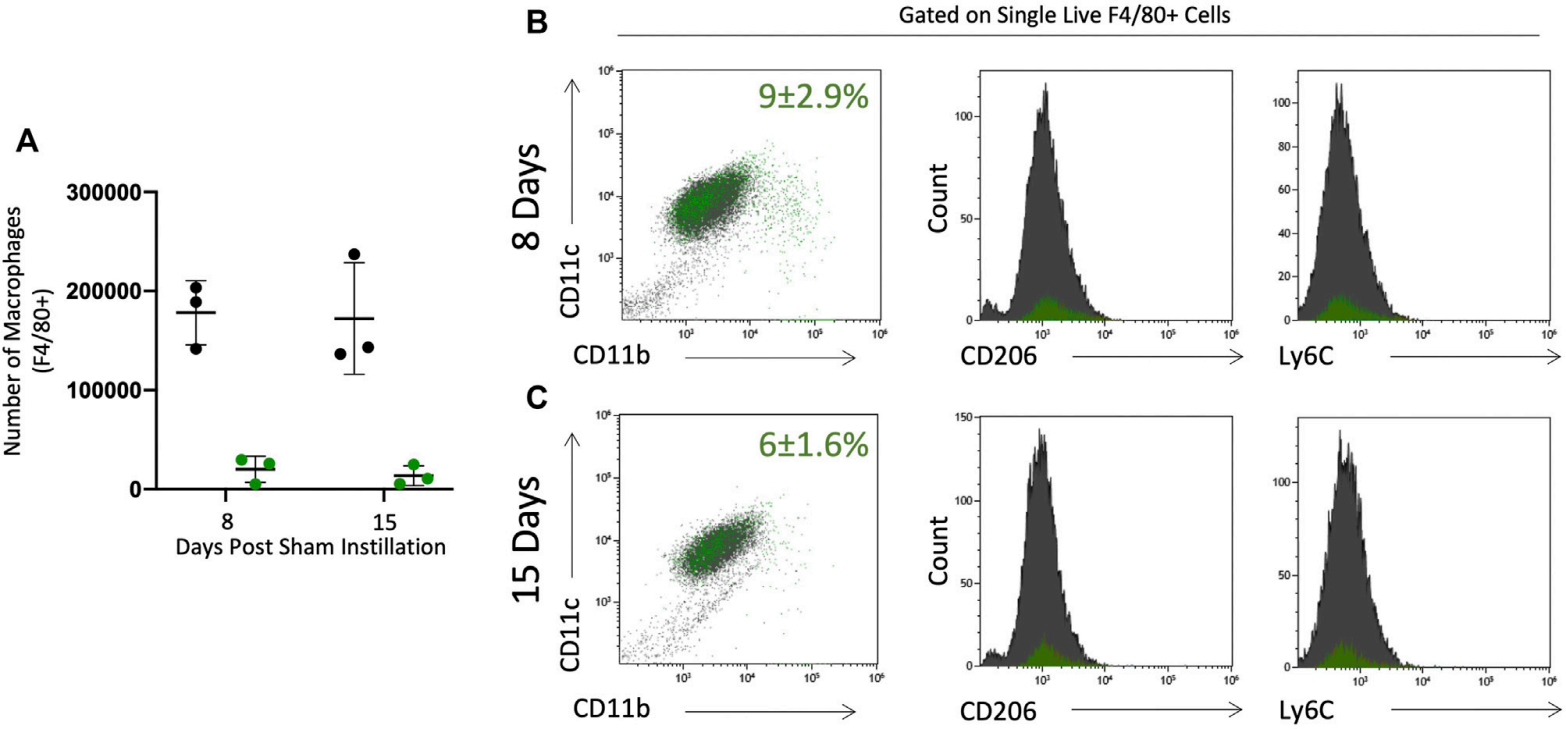
BAL macrophages collected from sham instilled GFP chimeric mice are consistent with resident alveolar macrophages. Pulmonary macrophages were collected by bronchoalveolar lavage in GFP chimeric mice 8 and 15 days post sham intratracheal instillation. Origin was determined by GFP expression to identify tissue resident (black) and monocyte originated (green) macrophages. Tissue resident (black) and monocyte originated (green) macrophages were quantified as absolute number **(A)**. Percent (± SE) of total F4/80 + single live cells that are monocyte originated (green dots) is given in CD11c vs. CD11b plots in panels B&C. Maturity (CD11c and CD11b expression) and phenotype (CD206 and Ly6C) were assessed by flow cytometry at 8 **(B)** and 15 **(C)** days post sham instillation. Tissue residency (black) or monocyte origination (green) did not alter maturity or activation marker expression.

Sham instilled mice were used to assess the effect of radiation on the lung and resident macrophage population. C57BL/6 mice were irradiated and bone marrow replaced with bone marrow from C57BL/6-Tg (CAG-EGFP)10sb/J. These mice then underwent intratracheal instillation process without instillation of solution (sham). Histologically mice were evaluated and there was no evidence of inflammation or structural alterations. Cells were harvested by lavage and phenotypically assessed by flow cytometry (Figure 1). Sham mice have a single population of pulmonary macrophages (F4/80+ CD11c+) that are resident (GFP-) to the lung (Figures 2B,C). Macrophages did not express Ly6C or CD11b and expressed low levels of CD206, congruent with alveolar macrophage phenotype (Bedoret et al., 2009; Guth et al., 2009; Watanabe et al., 2019). A small number of cells in the lavage were GFP+ 8 and 15 days post sham instillation (2.0 × 10^4^ and 1.4 × 10^4^, respectively) and their expression of subsequent markers are similar to GFP- macrophages (Figures 2B,C). The total number of GFP- macrophages remains constant with time following sham instillation (Figure 2A).

All protocols adhered to the NIH Guide for the Care and Use of Laboratory Animals and were approved by the Rutgers’ Institutional Animal Care and Use Committee.

### iNOS Inhibition

Diluent PBS, 1400 W (81520, Cayman Chemical Ann Arbor MI), was delivered by osmotic pump (2002, Alzet Cupertino,CA) subcutaneously implanted 3 days prior to intratracheal instillation. Mice studied at the 15 days time point were also intraperitoneally injected once daily for 4 days to continue past pump duration, and 1400 W was delivered at 10 mg/kg/h or a total dose of 60 μg as previously reported (Atochina-Vasserman et al., 2007).

### Intratracheal Instillation

Bleomycin (Sigma St. Louis MO), or diluent PBS, was intratracheally instilled in isoflurane anesthetized mice. Mice were treated with 3 units/kg bleomycin. Mice from each group (WT, 1400W, PKO, and BMKO) were instilled with PBS and evaluated at 8 and 15 days. Macrophage maturity and phenotype was similar between PBS instilled mice (Supplementary Figure S1).

### Sample Collection

Mice were sacrificed 8 and 15 days post intratracheal instillation by ketamine/xylazine overdose followed by exsanguination. Hearts were perfused with heparinized saline to clear red blood cells from lungs. Lungs were cannulated and bronchoalveolar lavage (BAL) was performed. Following BAL, left lungs were inflation fixed with 3% paraformaldehyde 2% sucrose, ethanol dehydrated, and paraffin embedded for sectioning.

### Cell Analysis

BAL was centrifuged to collect cell pellets and supernatant stored for protein measurements (Bradford assay). Cells were counted by Multisizer (Beckman Coulter Indianapolis IN). A total of 30,000 cells were spun for cytospin, stained with KWIK-DIFF (Thermo Scientific) and morphologically analyzed for cell type. A total of 200,000 cells (or remaining if insufficient) were immunostained for flow cytometry using the following antibodies: CD206-PE (Biolegend 141706), Ly6C-PerCP/Cy5.5 (Biolegend 128012), F4/80-PE/Cy5 (Biolegend 123114), CD11b-APC (Biolegend 101212), and CD11c-AF700 (Biolegend 117320). Manufacturer protocols were followed including 10-min Fc block (TruStain FcBlock Biolegend 101320) followed by 30-min antibody incubation at manufacturer suggested concentrations. Viability dye-eFluor780 (eBiosciences 65–0865–14 San Diego, CA) was incubated after antibodies for 30 min and washed out before paraformaldehyde fixing cells. Gallios flow cytometer (Beckman Coulter Indianapolis IN) was used to analyze cell fluorescent expression. Data was analyzed using Kaluza software, and all BAL cells were gated on F4/80 and viability to examine the macrophage population (Beckman Coulter Indianapolis IN). Gating strategy is shared in Figure 1.

### Tissue Analysis

Paraffin blocks of left lungs were sectioned for hematoxylin and eosin staining and immunohistochemistry. GFP (Abcam 290, 1:5000 primary dilution), CD11b (Abcam 133357, 1:3000 primary dilution), COX2 (Abcam 15191, 1:2000 primary dilution), CD206 (Abcam 64693, 1:500 primary dilution), and Ym1 (StemCell 01404, 1:500 primary dilution) antibodies were used to evaluate in tissue protein expression by IHC (secondary antibody 1:2000 [Vector Burlingame, CA]). Hematoxylin and eosin stained lung sections were scored for inflammation based on previously reported scoring system by two blinded reviewers (Rudmann et al., 1998). Scoring assessed peribronchial and perivascular epithelium hypertrophy, cellular recruitment, and alveolar septal defect. Images were taken on the VS120 microscope using Olyvia Software at 200x.

### Statistical Analysis

Data analyses were performed on GraphPad Prism 8. Parametric data were analyzed by 2-way ANOVA followed by *t*-test with Bonferroni correction. Mann-Whitney was used to analyze nonparametric data. Parametric data is expressed as mean and standard error of the mean, and nonparametric data as median. *p* value less than 0.05 was considered significant.

## Results

### The Tissue Resident Macrophage Population Is Preserved in Chimeric Mice

A panel of antibodies were used to phenotypically assess macrophage populations: the integrins CD11c, a protein expressed by pulmonary macrophages, CD11b, a migratory integrin, Ly6C, expressed by pro-inflammatory macrophages, and CD206, a scavenger receptor involved highly expressed in pro-resolution macrophages (Figure 1). Tissue resident macrophages (TRM) express F4/80 and CD11c with low expression of CD11b, Ly6C, and CD206. In sham instilled GFP chimeric mice, TRMs are the dominant cell type collected by bronchoalveolar lavage. Over 90% of macrophages collected 8 and 15 days post sham instillation are GFP- indicating they are tissue resident (Figure 2A). Of the F4/80 + cells, less than 10% are GFP+ either 8 or 15 days post sham instillation (Figures 2B,C first panel), indicating there is minimal monocyte recruitment to the lung lining. Macrophages in sham instilled mice express low levels of CD206 and Ly6C, regardless of GFP expression (Figures 2B,C). These data, along with the observation that over 92% of the bone marrow derived cells are GFP+ in this model (Figure 1), demonstrates that the presence of GFP within BAL macrophages can be used as a marker of cells that have originated from the bone marrow.

The effects of PBS instillation on macrophage phenotype were assessed in wild type mice (Supplementary Figure S1). Macrophages collected within the BAL are also consistent with mature TRM phenotype (F4/80+ CD11c+ CD11b-) (Supplementary Figure S1A). Small numbers of CD11c+ CD11b+ (intermediate) and CD11c- CD11b+ (immature) macrophage populations are present in all PBS instilled mice (Supplementary Figures S1B, S1C). There is no significant increase in CD206 or Ly6C expression (Supplementary Figure S1D). These data show that, as previously proposed (Savani et al., 2001), PBS instillation does represent a minor form of lung injury with minimal macrophage activation.

### ITB Induces a Loss of Tissue Resident Macrophages and Recruitment of Macrophages of Monocyte Origin

**FIGURE 3:**
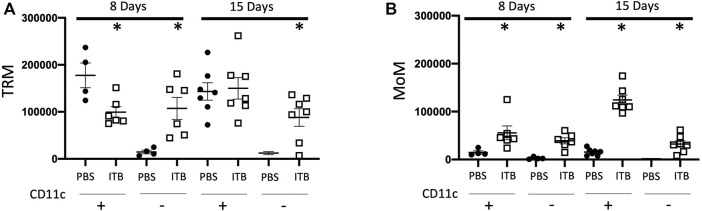
Tissue resident macrophages (TRM) and monocyte originated macrophages (MoM) are dynamic populations following instillation. **(A-B)** Gated on single live cells, TRMs are GFP- F4/80+ **(A)** and MoMs are GFP+ F4/80+**(B)**. CD11c expression further identifies macrophage phenotype. (*n* = 4–7 per group) *p < 0.05 compared to PBS.

Similar to the sham mice, the majority of BAL cells following PBS instillation are mature TRMs (GFP- F4/80+ CD11c+) at both 8 and 15 days. In contrast to PBS instilled mice, 8 days after ITB the percentage of BAL cells that are GFP- F4/80+ CD11c+ falls to 33%. They are largely replaced by immature TRMs (GFP- F4/80 + CD11c-). After 15 days there is a restoration of the mature TRMs to 40% of the total BAL cells. Importantly, although the percentage of BAL cells that are mature TRMs is still only 40%, the absolute number is close to that in PBS treated mice (15 ± 2.3 × 10^4^ vs 14 ± 1.8 × 10^4^) (Figure 3A). These data demonstrate that there is a loss of TRMs following ITB but that the resident pool is capable of regeneration.

Following PBS instillation, there is no recruitment of MoMs (GFP+ F4/80+) irrespective of maturity as denoted by CD11c expression. However, in conjunction with the loss of TRMs, following ITB, there is a significant recruitment of MoMs as indicated by the increase in GFP+ cells in the BAL (8.5% in PBS at 8 days as compared to 31.3% in ITB at 8 days). This recruitment appears to be progressive as 15 days post ITB the number of GFP+ F4/80+ CD11c+ cells increases from 5 ± 1.4 × 10^4^ at 8 days post ITB to 12 ± 0.9 × 10^4^ at 15 days (Figure 3B).

### ITB Induces CD206 Expression in Tissue Resident Macrophages

**FIGURE 4:**
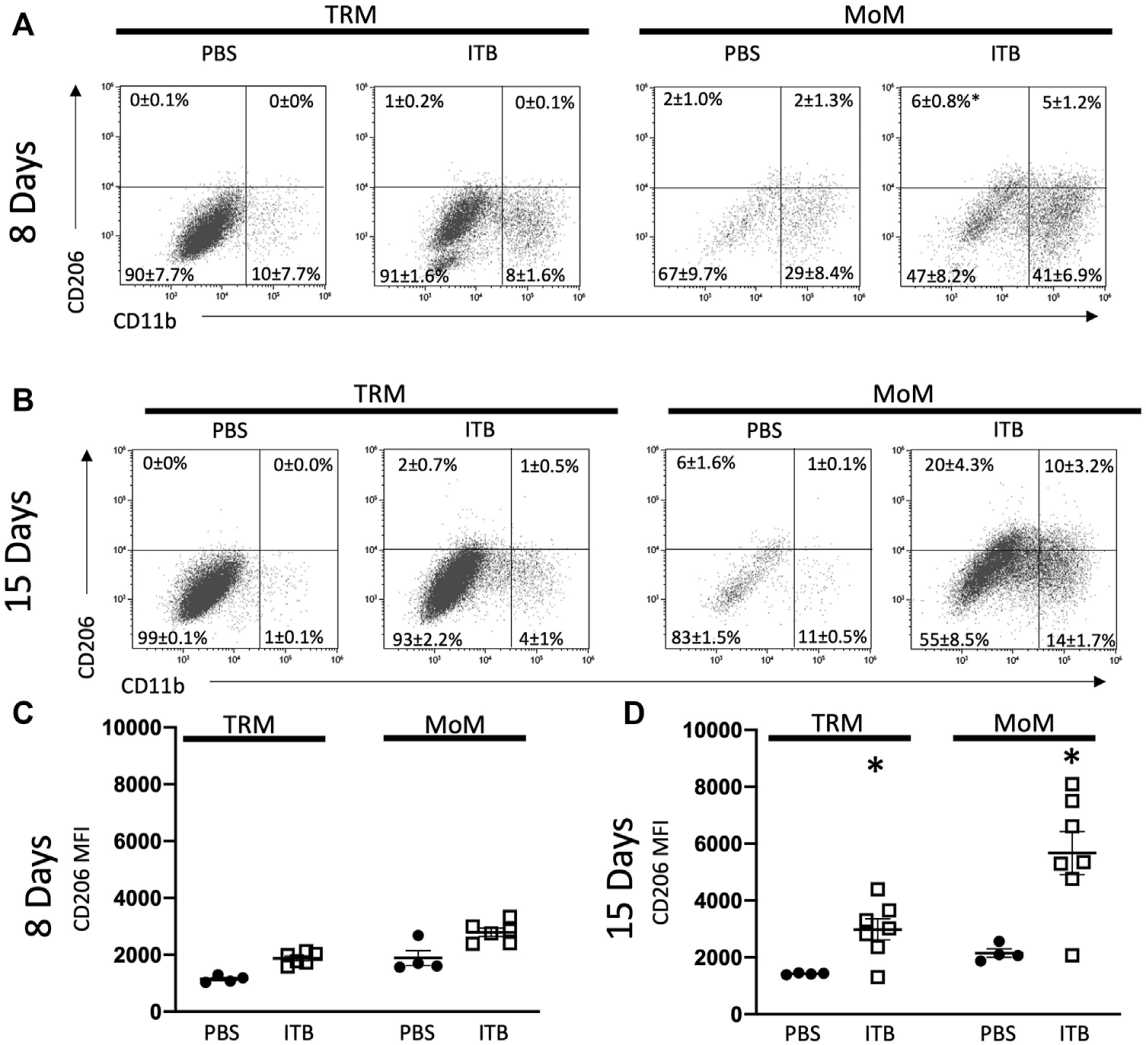
TRM and MoM populations increase CD11b and CD206 expression post ITB. **(A–B)** TRM and MoM expression of CD206 and CD11b at 8 **(A)** and 15 **(B)** days post instillation in representative plots. The mean percentages ±SE for each quadrant are reported. **(C–D)** CD206 expression reported as mean fluorescence intensity (MFI) of TRM and MoM populations at 8 **(C)** and 15 **(D)** days post ITB. (*n* = 4–7 per group) *p < 0.05 compared to PBS.

Having established that there is a loss of TRMs upon ITB treatment that appears to be restored with time, we examined whether there was an alteration of phenotype within these expanding cells. Therefore, we examined the expression of CD206 and Ly6C as well as CD11b in mature TRMs (GFP- F4/80+ CD11c+) within the BAL. As the expression of CD206 occurs along a continuum, we examined it by mean fluorescence intensity (MFI). Ly6C is either expressed as high or low on macrophages, and thus we report the percent of macrophages expressing high and low levels of Ly6C. Interestingly, CD11b expression was not increased within TRMs following ITB (Figures 4A,B). CD206 expression within TRMs was not dependent upon CD11b expression. In TRMs there was a significant increase in CD206 expression at 15 days, but not 8 days, after ITB relative to PBS (Figures 4C,D). This contrasts with Ly6C, whose expression is not observed in this population of cells (data not shown). This is consistent with the role of TRMs as reparative within the lung following injury (Watanabe et al., 2019).

### ITB Increases MoM Activation and Maturation

**FIGURE 5:**
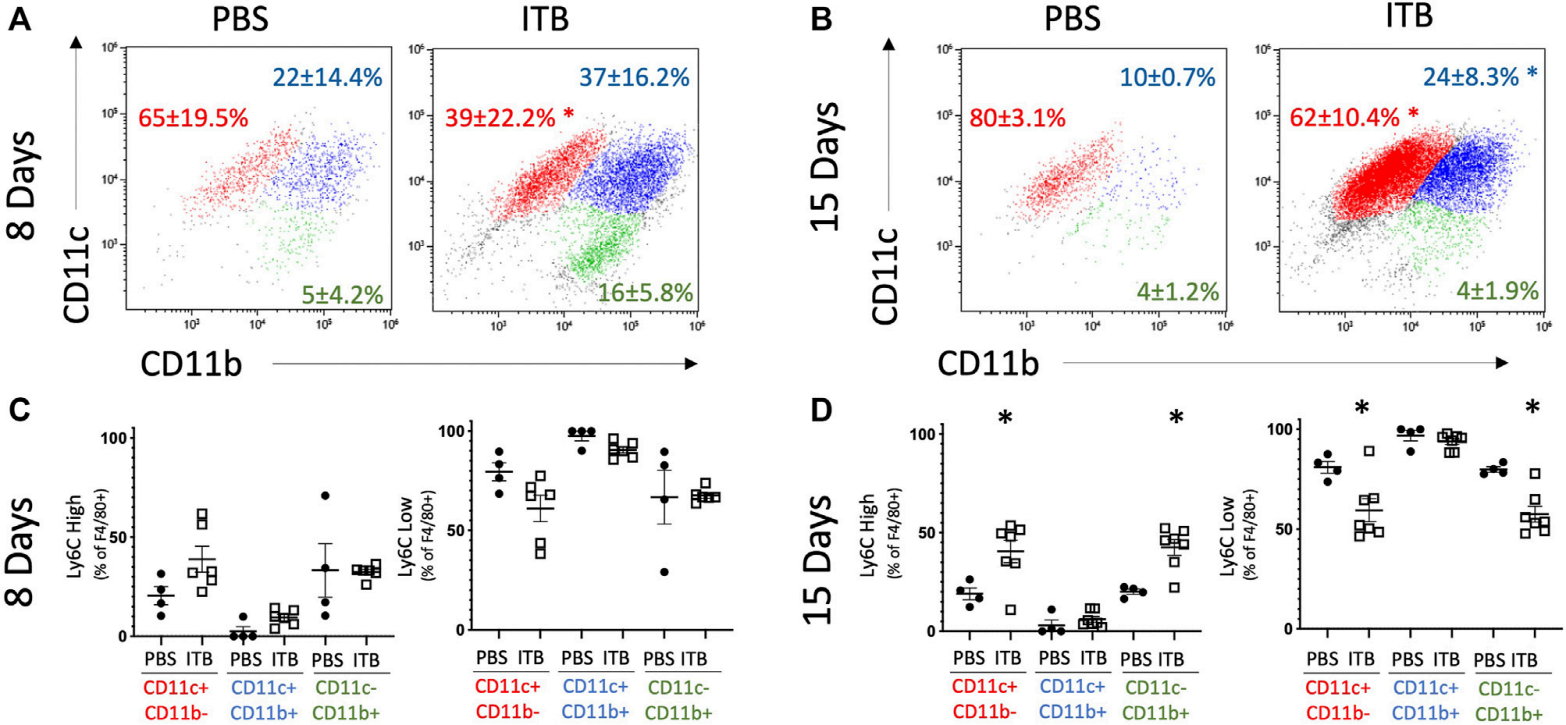
MoMs are recruited to the lung and increase Ly6C expression following ITB. **(A-B)** CD11c and CD11b expression on MoMs demonstrate 3 distinct populations at 8 **(A)** and 15 **(B)** days post instillation. **(C–D)** Ly6C expression is gated as either high or low in MoMs collected at 8 **(C)** and 15 **(D)** days post instillation. (*n* = 4–7 per group) *p < 0.05 compared to PBS.

MoMs (GFP+ F4/80+ cells) also express an increased level of CD206, as well as CD11b, relative to PBS control at 15 days (Figure 4D). MoMs were examined for CD11b and CD11c expression. Under all conditions, the macrophages can be clearly divided into CD11c+ CD11b- (mature, red); CD11b+ CD11c- (immature, green); and CD11b+ CD11c+ (intermediate, blue). In accordance with the increase in MoMs that occurs with ITB (Figure 3), one can see an increase in CD11b expression in CD11c+ population at 15 days post injury (Figures 5A,B). At 15 days post ITB there is a significant increase in the percent of Ly6C High immature and mature MoMs (Figure 5D). These data indicate that within the lung lining MoMs can take on both an inflammatory (Ly6C High) and a reparative (increased CD206) role.

**FIGURE 6:**
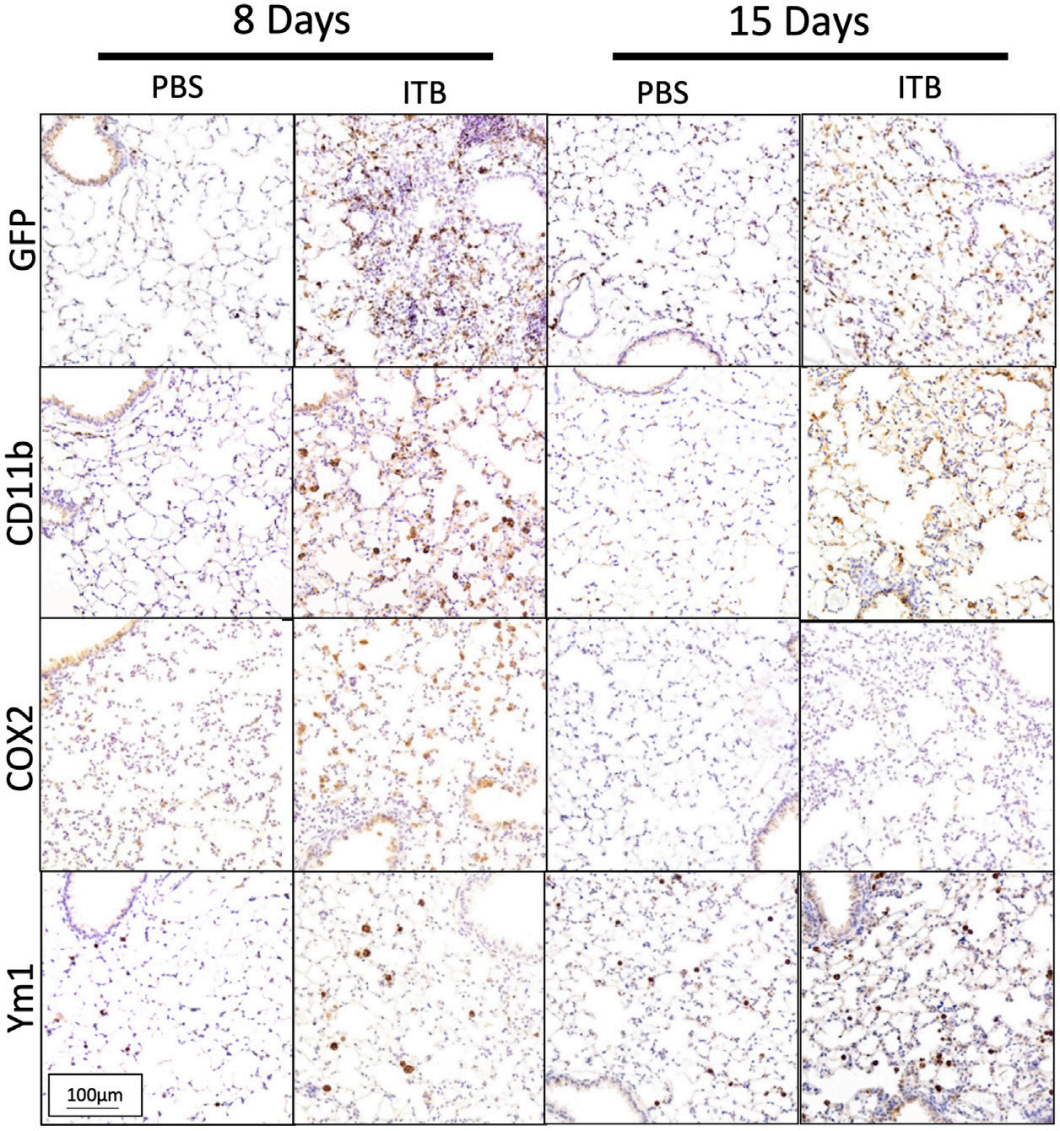
Macrophages are recruited and activated in lung. Lungs post lavage are assessed histologically for macrophage recruitment and activation. **(A)** Lungs collected from PBS and bleomycin instilled mice are stained for GFP, CD11b, COX2, and Ym1. Representative pictures are at 200x. (*n* = 4–7 mice per group)

The previous data have all examined macrophages within the BAL, which do form over 90% of the BAL cells post ITB. However, in order to examine the effect of ITB on TRMs and MoMs within the lung tissue we used a histological approach (Figure 6). Immunohistochemistry of post ITB lungs shows an increase in GFP staining within the lung parenchyma 8 days after ITB. GFP+ cells are predominantly in areas of perivascular and peribronchial infiltration. Compared to 8 days post ITB, at 15 days there is less GFP. CD11b staining at 8 days post ITB also increases relative to PBS especially within the lung parenchyma. However, at 15 days post ITB, while there are still focal points of staining, much of the CD11b staining is non-cellular, possibly as a result of release from recruited macrophages. COX2, often considered a marker of acute inflammatory macrophage activation (Sunil et al., 2012), is more prevalent at 8 days post ITB but not 15 days. Ym1, which encodes the enzyme chitinase, and is often considered a marker of alternative activation within macrophages, is expressed as early as 8 days post ITB. However, at 15 days, while the distribution of expression is not altered, staining is visibly darker.

### Pan iNOS Inhibition Alters Response to ITB

**FIGURE 7:**
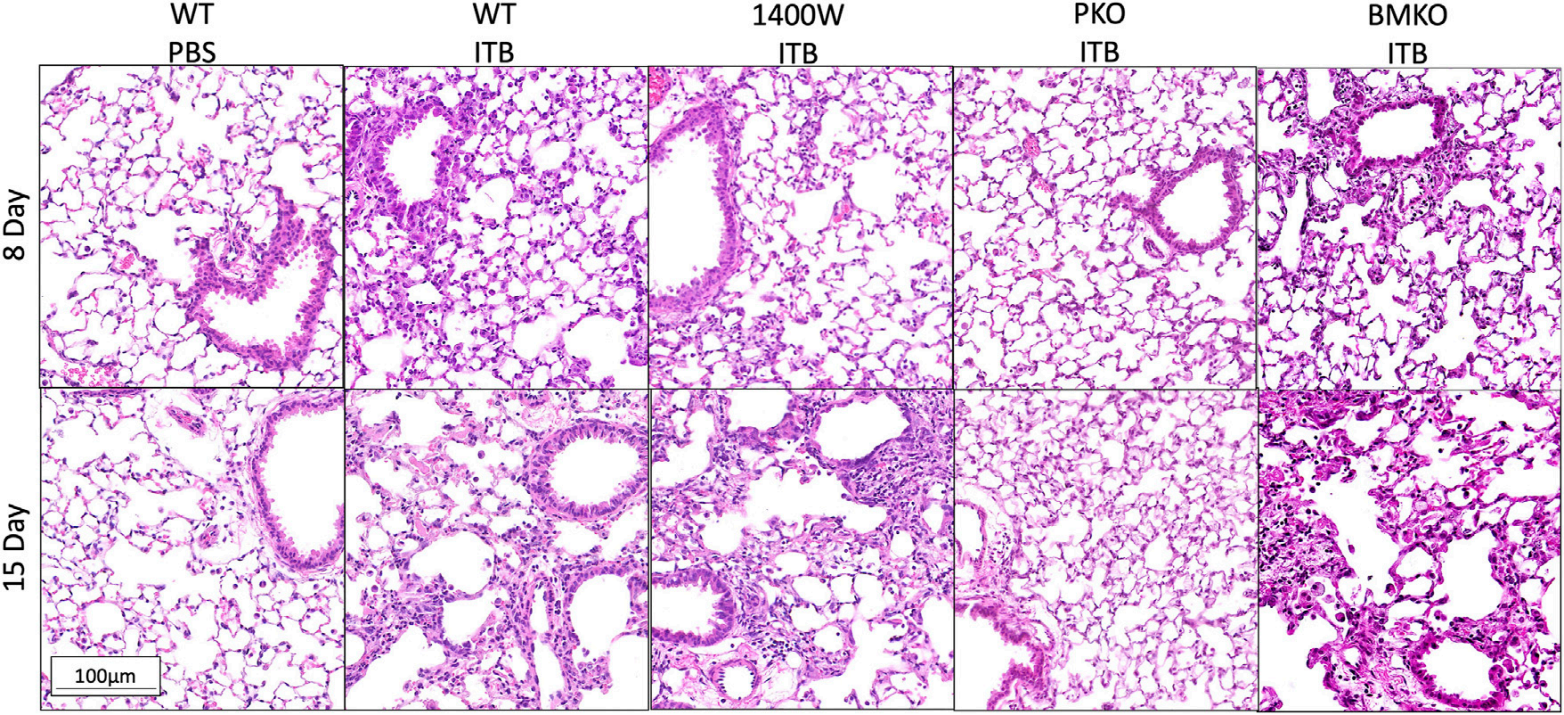
Lungs collected post lavage were stained by H&E and morphology assessed. Representative images are shown for each group. Representative pictures are at 200x. (*n* = 3–7)

**FIGURE 8:**
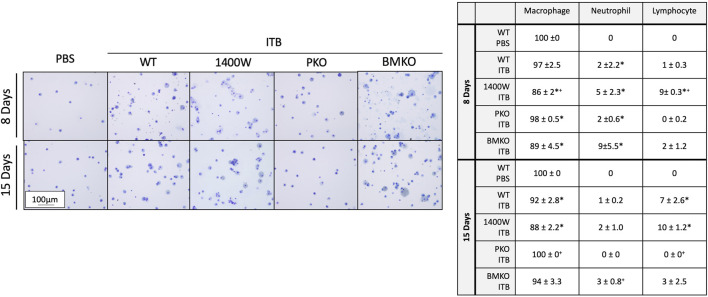
BAL cells include macrophages, neutrophils, and lymphocytes. Cells collected by bronchoalveolar lavage were assessed by morphology and size to identify cell type. Macrophages, neutrophils, and lymphocytes were identified and quantified. (*n* = 3–7 per group) *p < 0.05 compared to PBS, + *p* < 0.05 compared to ITB.

Previously we have observed that iNOS inhibition reduces inflammation early in response to ITB, but exacerbates fibrosis (Guo et al., 2016). We reasoned that iNOS may play differential roles in TRMs and MoMs. Therefore, we examined the effects of systemic iNOS inhibition using 1400 W on macrophage activation following ITB. In accordance with our previous findings 1400 W had a small effect on pulmonary inflammation at day 8 post ITB as shown by histological assessment (Figure 7; Table1). However, over time, injury worsens in 1400 W treated mice as reflected by an increase in the inflammatory score at 15 days. Examination of the BAL cells by cytospin reveals that the predominance of the recruited cells are macrophages, although there is a significant increase in lymphocyte recruitment at 8 and 15 days in the presence of 1400 W (Figure 8).

**TABLE 1:** Inflammation and injury were assessed following instillation of PBS and bleomycin. Inflammation score was blindly assigned based on histological assessment. Protein and total cell number was measured in bronchoalveolar lavage fluid. (*n* = 3–7 per group) *p < 0.05 compared to PBS, + *p* < 0.05 compared to ITB.

		PBS	ITB	1400 W	PKO	BMKO
8 Days	Inflammation Score	0	3	1	1	2
	BAL Protein (μg/ml)	94.9 ± 4.3	2,067 ± 94.3*	2044 ± 148.9*	829 ± 375.7	1,352 ± 578.6*
	BAL Cell Number (x10^4^)	22 ± 2.8	47 ± 4.0*	39 ± 3.3*	33 ± 5.3	29 ± 6.6^+^
15 Days	Inflammation Score	0	3*	3.5	1^+^	2
	BAL Protein (μg/ml)	118.2 ± 33.0	2,067 ± 115.9*	2045 ± 137.8*	739 ± 67.7^+^	1,976 ± 176,3*
	BAL Cell Number (x10^4^)	19 ± 1.0	64 ± 3.0*	64 ± 3.3*	38 ± 9.0^+^	47 ± 5.3*

### iNOS Location Is Critical to the ITB Response

iNOS is inducibly expressed in times of inflammation by both myeloid derived and epithelial cells (Wong and Billiar, 1995). Therefore, there are several potential sources of NO following ITB. iNOS chimeras were created in order to study the role of recruited and pulmonary derived iNOS in macrophage activation following ITB. Pulmonary knockouts (PKO) were created by replacing the bone marrow of *NOS2* ^*−/−*^ mice with WT cells such that iNOS could be expressed by MoMs but not TRMs or pulmonary epithelial cells. Bone marrow knockouts (BMKO) were created by replacing the bone marrow of WT mice with cells from *NOS2* ^*−/−*^ mice thereby the MoMs are iNOS incompetent and pulmonary derived iNOS preserved*.* In PKO the response to ITB was significantly attenuated. There were minimal signs of inflammation or injury at the histological level at both 8 and 15 days post ITB (Figure 7). There was no increase in BAL cell number at 8 or 15 days compared to PBS instilled mice and increases in BAL protein content were limited in PKO (Figure 7; Table 1). In contrast, the BMKO mice had milder inflammation 8 days post ITB, evidenced by attenuated cellular recruitment though similar BAL protein content (Table 1). However, ITB response was similar in BMKO mice at 15 days (Table 1). Interestingly, macrophages from BMKO mice appear enlarged and to display evidence of engulfment of other cells (Figure 8). Neutrophilia also persists until 15 days post ITB in BMKO.

### iNOS Location Is Critical to Determining the BAL Macrophage Activation Response to ITB

For the purposes of this study the more critical question is how does loss of iNOS function alter macrophage recruitment and activation to ITB. Both our 1400 W and our PKO experiments were performed with GFP+ bone marrow cells allowing for identification of TRMs and MoMs. However, in the BMKO experiments the myeloid cells were *NOS2* ^*−/−*^ but not GFP+; therefore, GFP could not be used to differentiate TRMs and MoMs. As iNOS may play a homeostatic role, we carefully examined the effect of PBS in all iNOS manipulated mice. Baseline macrophage phenotype was similar in all and consistent with TRM phenotype (Supplementary Figure S1).

**FIGURE 9:**
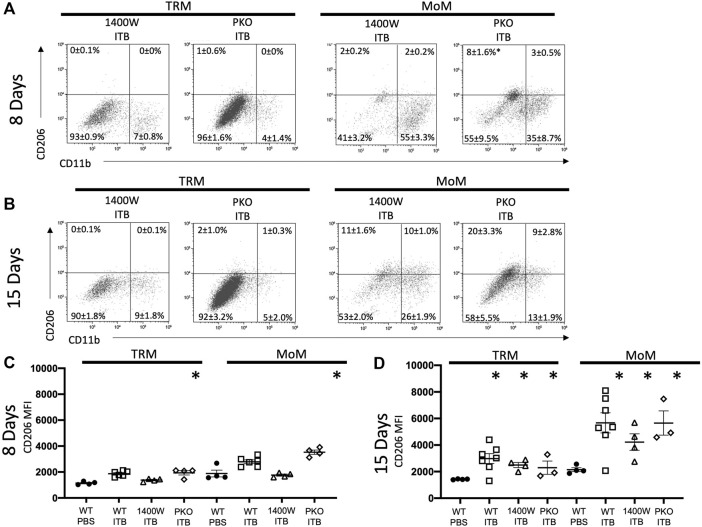
Systemic iNOS inhibition and pulmonary specific iNOS knockouts have an altered response to ITB. Following bleomycin instillation, macrophages were assessed from 1400 W treated and PKO mice. **(A–B)** Macrophages were identified as TRMs and MoMs based on GFP expression 8 **(A)** and 15 **(B)** days post instillation in single live F4/80 + cells. The mean percentage ±SE for each quadrant is reported. **(C–D)** CD206 expression is reported as mean fluorescence intensity (MFI) 8 **(C)** and 15 **(D)** days post instillation. (*n* = 3–7 per group) *p < 0.05 compared to PBS, +<0.05 compared to ITB.

**FIGURE 10:**
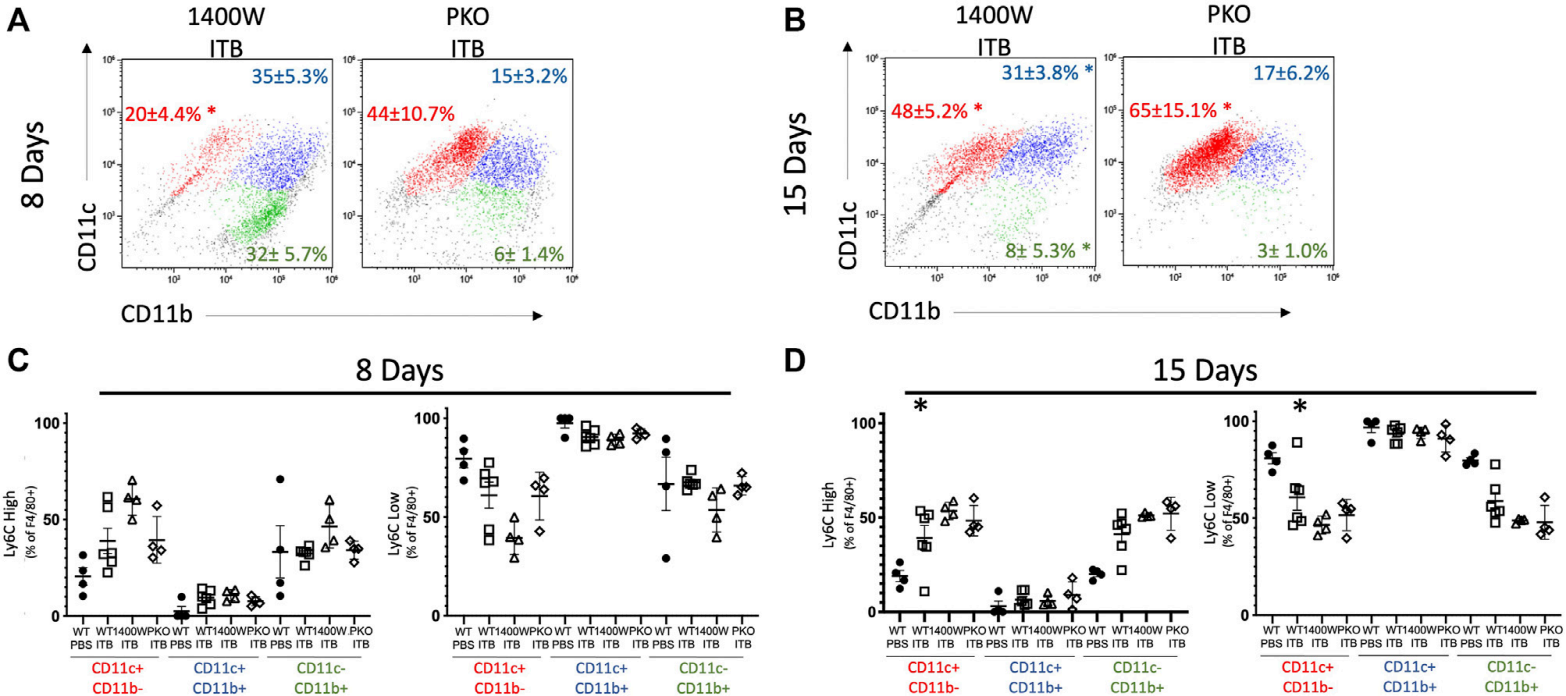
Systemic and pulmonary loss of iNOS alters MoM response to ITB. MoM maturity and proinflammatory activation following ITB in 1400 W treated and PKO chimeras. **(A–B)** MoM expression of CD11c and CD11b 8 **(A)** and 15 **(B)** days post ITB. **(C–D)** Ly6C expression is gated as either high or low at 8 **(C)** and 15 **(D)** days post ITB. (*n* = 3–7 per group) *p < 0.05 compared to PBS, + *p* < 0.05 compared to ITB.

As with ITB alone (Figures 4A,B), irrespective of iNOS competency, there was minimal CD11b expression within TRMs at 8 or 15 days post ITB (Figures 9A,B). However, at 15 days post ITB, MoMs collected from 1400 W treated mice showed an increase in CD11b expression, similar to ITB alone. In particular, the CD11b+ CD11c- MoM population is expanded in 1400 W treated mice compared to ITB alone (32 ± 5.7% vs 16 ± 5.8%) at 8 days. This increase is not seen in PKO mice, and in fact the dominant MoM population in PKO mice is CD11c+ CD11b- (44 ± 10.7%) (Figure 10A). PKO mice experience a unique increase in CD206 expression in TRMs and MoMs at 8 days following ITB compared to PBS treated mice (Figure 9C). At 15 days, MoMs and TRMs increase CD206 expression irrespective of iNOS competency (Figure 9D).

As stated previously, Ly6C is not detected in TRMs but is observed in mature MoMs (GFP+ F4/80+ CD11c+) following ITB. At 8 days following ITB, mice treated with 1400 W have a further increase in the percent of Ly6C high macrophages (Figure 10C). At 15 days post ITB, the percent of Ly6C high macrophages is elevated in all ITB groups compared to PBS.

**FIGURE 11:**
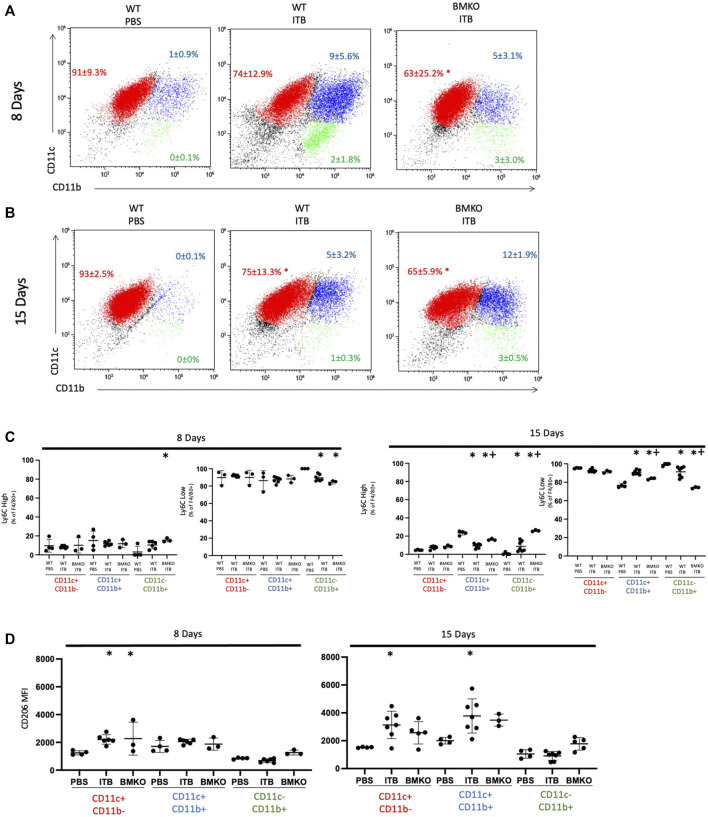
Macrophage activation is influenced by loss of iNOS in bone marrow derived cells. Macrophage maturity and activation in wild type and BMKO mice following ITB. **(A–B)** CD11c and CD11c expression on single live F4/80 + cells collected by bronchoalveolar lavage 8 **(A)** and 15 **(B)** days post ITB. **(C–D)** Expression of activation markers Ly6C **(C)** and CD206 **(D)** on sub-populations of macrophages. (*n* = 3–7 per group) *p < 0.05 compared to PBS + *p* < 0.05 compared to ITB.

We cannot differentiate between tissue derived and recruited cells in BMKO mice; therefore, we analyzed macrophage expression of Ly6C and CD206 as a function of maturity (based on CD11c and CD11b expression) (Figure 11). BMKO persistently reduced the overall maturity of the macrophage population as evidenced by a decrease in the number of CD11c+ CD11b-macrophages at 8 (63 ± 25.2%) and 15 (65 ± 5.9%) days post ITB (Figures 11A,B). At 8 days post ITB, there is an increase in CD206 expression by CD11c+ CD11b-macrophages following ITB regardless of iNOS function. At 8 and 15 days, the percent of Ly6C high CD11c- CD11b+ macrophages was increased in BMKO, compared to PBS and WT ITB (Figure 11C). BMKO mice fail to increase CD206 expression following ITB (Figure 11D). These data indicate that bone marrow derived iNOS is involved in macrophage maturation and the expression of alternative activation markers in ITB.

### iNOS Location Is Key in Regulating Macrophage Recruitment and Activation in the Lung Tissue

**FIGURE 12:**
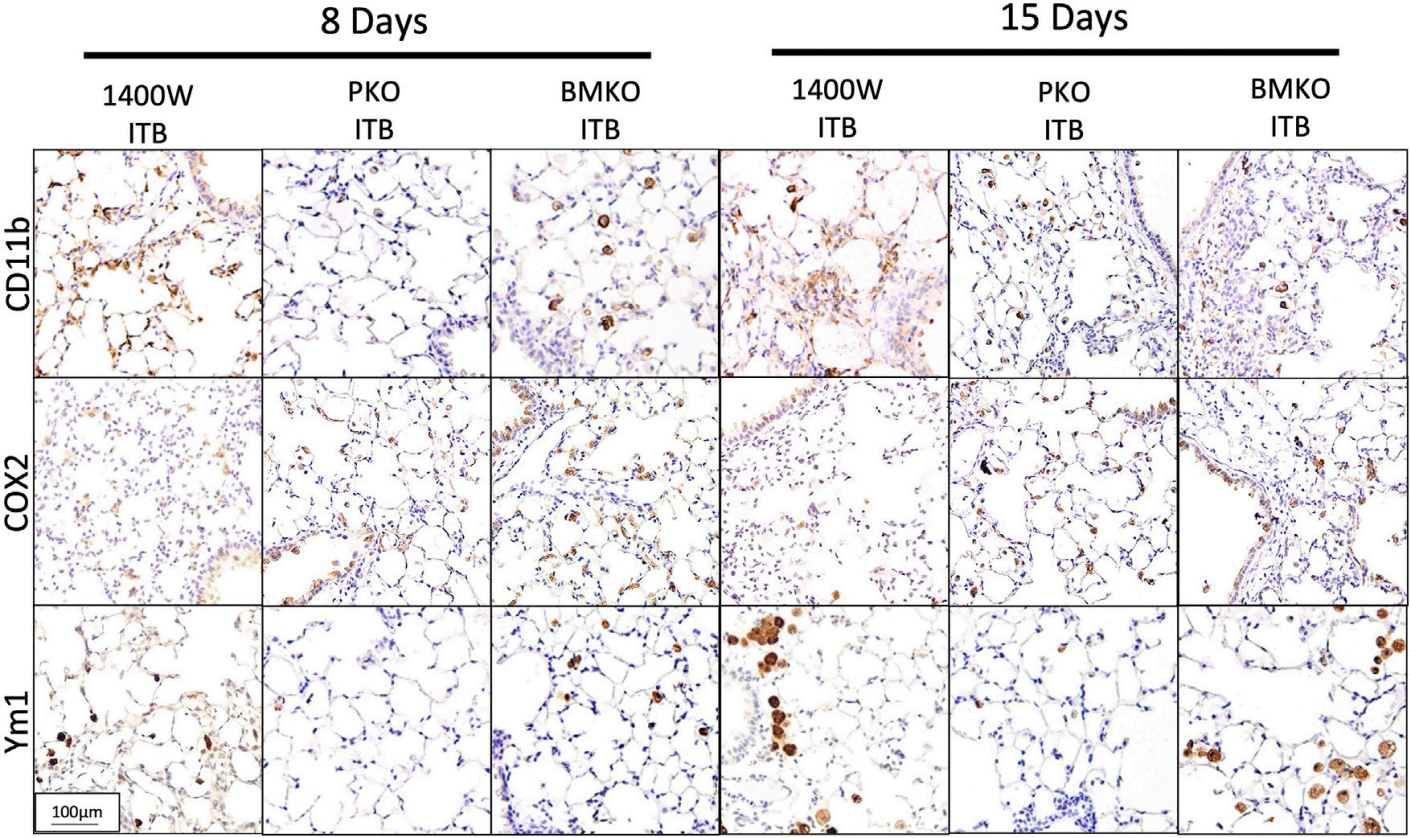
Macrophage activation in lung tissue differs based on iNOS competency. Lungs collected from bleomycin instilled mice also treated with 1400 W or iNOS chimeras are stained for CD11b, COX2, and Ym1. Representative pictures are at 200x. (*n* = 4–7 per group).

Previously (Figure 6A) we showed that with ITB there was CD11b staining within the lung 8 days post ITB and that this was accompanied by COX2 at 8 days and YM-1 at 15 days post injury. Examination of the lung tissue following ITB in iNOS compromised mice reveals the importance of pulmonary-derived NO production (Figure 12). COX2 staining is less evident with 1400 W treatment at 8 days post ITB, but Ym1 staining more obvious at 15 days. Loss of bone marrow derived iNOS (BMKO) does not affect staining at 8 days, but shows a persistent COX2 and Ym-1 stain at 15 days post ITB. CD11b staining was evident in both 1400 W inhibited and BMKO mice. In PKO mice, all three stains were less evident than wild type ITB, indicating reduced recruitment and activation of macrophages in the lung tissue.

## Discussion

**FIGURE 13:**
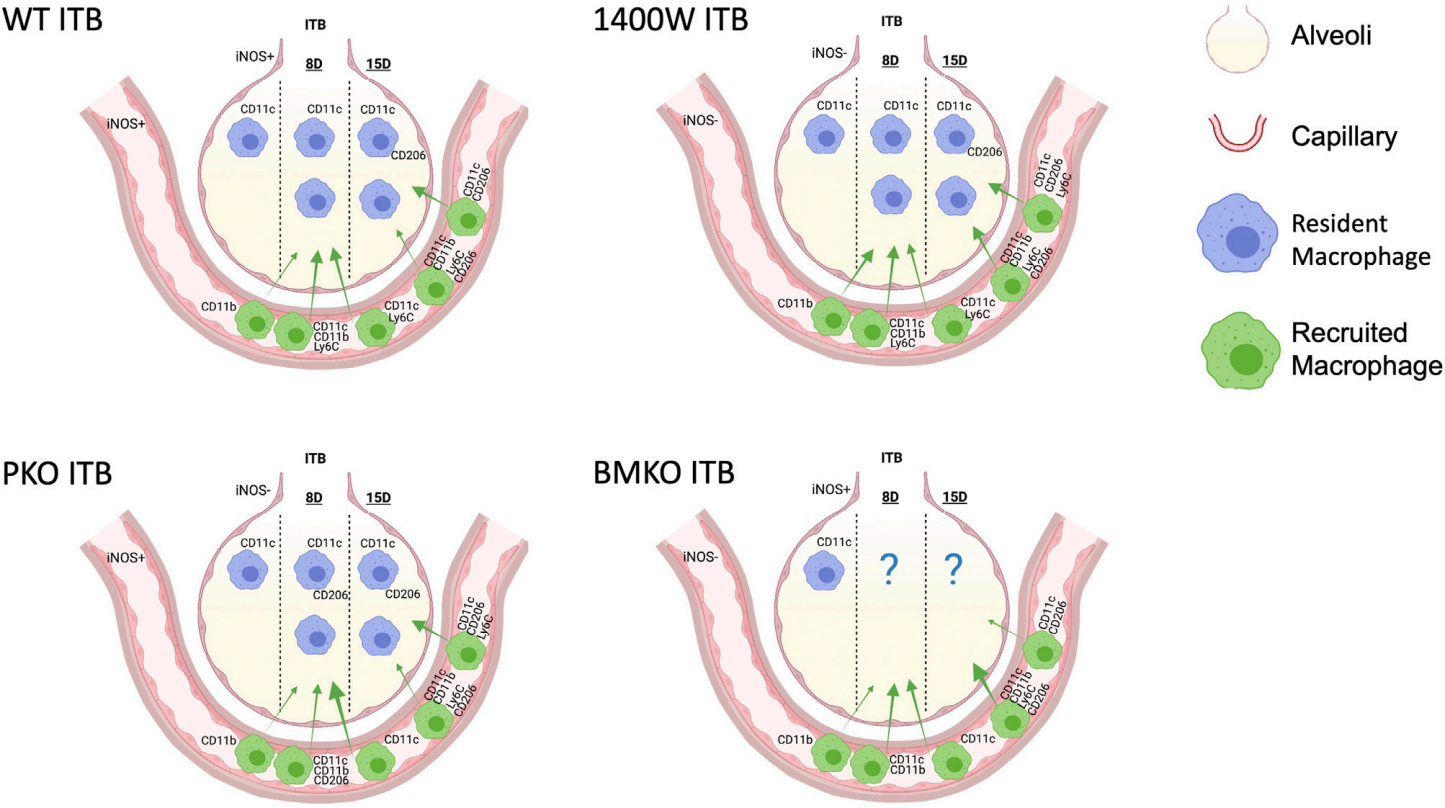
A model to illustrate the macrophage populations 8 and 15 days following intratracheal instillation of bleomycin (ITB) in wild type mice, those treated with 1400 W, and iNOS chimeras. Macrophage populations vary in expression and relative distribution (demonstrated by arrow size) in response to iNOS competency. Created with Biorender.com.

In this study we have examined how macrophage origin affects activation and function within ITB mediated ALI. Using a chimeric mouse model, we have examined the effects of injury on both resident (TRMs) and recruited (MoMs) macrophages. Further, we have identified the role that the physical location of iNOS plays in regulating its function in the context of inflammation and resolution within ITB mediated ALI. These data show that TRMs are lost early in the response to ITB but are capable of regenerating and that MoMs are recruited as early as day 8. In addition, TRMs are capable of activation but only towards an M2 phenotype, while MoMs are activated across the spectrum. Finally, iNOS activation within the lung is a critical determinant of both resident and recruited macrophage responses to ITB and in determining inflammatory outcome. The effects of iNOS activation we have observed within the ITB model are summarized in Figure 13.

In agreement with prior lung injury studies (Maus et al., 2004; Maus et al., 2006), our study demonstrates that acutely there is a loss of TRMs in response to ALI-induced by ITB (Figure 3). It is important to note that TRMs have a clear phenotype in that they display markers of maturity (F4/80 and CD11c), but not of activation (Ly6C and CD206). Interestingly, this population expands between 8 and 15 days post ITB showing that TRMs are capable of regenerating. In accordance with the concept of TRM expansion, a significant number of these newly appearing cells are CD11c, indicating that they are immature. In terms of activation, TRMs are capable of expressing CD206. At no point do we observe significant expression of Ly6C in this population; however, following ITB there is a significant increase in CD206. These data appear to indicate that TRMs function in the reparative mode within the lung but probably cannot adopt a cytotoxic function.

Similarly, to the TRMs, we do observe significant CD206 expression within the MoMs; however, they are also capable of expressing Ly6C. We observe an increase in the percent of Ly6C High within mature (CD11c+ CD11b-) and immature (CD11c- CD11b+) 15 days following ITB. Interestingly, this indicates there is still cytotoxic activation of macrophages at this later resolution stage.

The use of radiation was necessary to create chimeric mice but represents a potential limitation. The dose used in these experiments did not itself cause injury to the lung as evidenced by normal pulmonary histology in sham instilled mice. Radiation also has the potential to affect alveolar macrophages. Several reports suggest alveolar macrophages are resistant to radiation (van oud Alblas and van Furth, 1979; Tarling et al., 1987; Kennedy and Abkowitz, 1998; Landsman and Jung, 2007; Hashimoto et al., 2013). While others have used lung shielding (Janssen et al., 2010), we were limited to whole body irradiation as the length of time required to deliver 12 Gy x-ray radiation exceeds the duration of anesthetics. Despite whole body radiation, BAL cells collected from sham instilled mice are consistent with the phenotype of alveolar macrophages, CD11c+ CD11b- CD206 Ly6C low (Guth et al., 2009; Zaynagetdinov et al., 2013). It would appear, therefore, that this approach to creating chimeric mice allowed for assessment of cell origin’s influence.

Additionally, the experimental design is potentially limited by incomplete reconstitution of the bone marrow. While the percent of cells isolated from the bone marrow that are donor (GFP+) is over 92%, there is still evidence of recipient cells after 6 weeks of reconstitution. This demonstrates the potential for recruited cells to be GFP- and incorrectly identified as resident. Although the relative proportion of GFP- cells in the bone marrow is very low, it should be considered when using GFP- as a marker of residency.

The activation of macrophages following ITB has been extensively studied (Cavarra et al., 2004; Gasse et al., 2007). However, only recently has the role of cell origin in macrophage activation been proposed (Mould et al., 2017; Zhou and Moore, 2018). In the LPS model of ALI, Mould et al. measured the transcriptome of resident and recruited macrophages following LPS and found origin influenced activation (Mould et al., 2017). Alveolar macrophages are embryonically derived (Hashimoto et al., 2013), long lived (van oud Alblas and van Furth, 1979), and potentially self-replicating (Tarling et al., 1987; Hashimoto et al., 2013). Alveolar macrophages are influenced by their local environment and express proteins in line with M2 polarization at baseline (Guth et al., 2009), whereas monocyte derived macrophages arise in the bone marrow and are recruited to the tissue by cytokines. Though both populations are macrophages, the environments in which they reside have a great deal of influence on their phenotype and activation potential (Evren et al., 2020).

Our study supports the notion that cell origin is a major influencer in macrophage activation during the stages that follow ALI. Here we demonstrate TRMs only activate towards M2 polarization. However, MoMs are able to activate along a spectrum of M1 and M2 polarization. In fact, there is considerable overlap of M1 and M2 polarization in that cells can express either Ly6C or CD206, or both during the stages that follow ITB. Further characterization of these populations using approaches that allow for more in depth evaluation of the activation spectrum and complexity will shed light on the many roles MoMs play during ALI.

As mentioned previously, iNOS inhibition appears to have both beneficial and negative effects in the context of ALI (D’alessio et al., 2012; Guo et al., 2016). The data presented here appear to show that cellular origin affects phenotypic outcome, as well as influences the effects of iNOS function. An important proviso here is that we have not specifically measured iNOS function through a technique such as nitrite production. Rather we have observed the effects of loss of iNOS from specific cells. It is important to remember that genetic ablation is not the same as enzyme inhibition as within the first there is the possibility of compensatory changes, while within the latter there is always the question of efficiency of inhibition. Bearing this caveat in mind we examined location specific deletion of iNOS compared to systemic inhibition in terms of altering macrophage activation and recruitment.

In mice treated with 1400W, the response to ITB was exaggerated at 8 days in that there was increased recruitment to the lung of CD11b+ macrophages and lymphocytes. The percent of Ly6C high MoMs was increased at 8 days post ITB in 1400 W treated mice. There is also an increase in the number of Ly6C high macrophages at 8 and 15 days post ITB in BMKO mice. Due to the inability to identify cell origin in the BMKO mice, it is impossible for us to definitively say if the lack of activation was in the TRM or MoM populations. However, due to the knowledge gained in the GFP chimera studies, we can assume that the gain of Ly6C high macrophages in BMKO mice is indeed in the MoM population. However, the reduction of CD206 in the CD11c+ CD11b- and CD11c+ CD11b+ populations at 15 days could be in either MoMs, TRMs, or both. In PKO mice both pulmonary epithelial cells and TRMs lack iNOS. Following ITB, PKO mice have reduced inflammation, recruitment, and activation. Interestingly, MoMs adopt a M2 phenotype, instead of M1 phenotype, 8 days post ITB. iNOS appears therefore to play a significant role in activation of the cell in which iNOS is expressed. These studies also suggest pulmonary derived iNOS is detrimental to the resolution of ALI.

While the studies we have performed demonstrate the importance of iNOS in driving the macrophage response to ALI, they do not provide evidence as to the molecular mechanisms involved. Several mechanisms by which loss of functional iNOS could alter macrophage activation are possible. NO is known to modify proteins such as NFκB (Kelleher et al., 2007), GAPDH (Hara et al., 2005), Keap1 (Buckley et al., 2008), SP-D (Guo et al., 2008), and Hmgb1 (Tsoyi et al., 2010) via S-nitrosylation. Such protein modifications influence inflammation and resolution pathways. NO also has the potential to influence macrophage activation by directly promoting glycolysis (Erusalimsky and Moncada, 2007), the energy pathway of classically activated macrophages (Kelly and O’neill, 2015). These NO dependent modifications require evaluation to elucidate the mechanism by which NO activates macrophages in ITB-mediated ALI.

It is also possible iNOS produces superoxide following ITB. iNOS requires the cofactor tetrahydrobiopterin (BH_4_) in order to produce NO (Hurshman et al., 1999). Without BH_4_ the enzyme can become uncoupled and produces superoxide (Stroes et al., 1998; Mcneill et al., 2015). Oxidized biopterins also act as a competitive inhibitor of NOS enzymes (Vasquez-Vivar et al., 2002; Crabtree et al., 2009). Oxidative stress is a known contributor to ITB mediated ALI. Therefore, it is possible iNOS is not functioning to produce NO but instead is uncoupled and producing superoxide. The loss of iNOS in the various chimeras may in fact reduce production of reactive oxygen species, and the resultant effects are not due to the reduction in NO production.

ALI is a complex pathology that often results in a failure to properly resolve leading to fibrosis, as in the ITB mediated model. This study identifies macrophage populations responsible for the stages following injury and their dependence on iNOS for activation. In particular, it points to pulmonary derived iNOS as the culprit in exaggerated and detrimental responses to ALI following ITB. That the location of iNOS expression is critical to its signaling function is interesting when one considers the role of NO as a diffusible second messenger. These studies highlight that the effects of NO generation may be specific to the originating cells. In other words, cell signaling in response to NO is dependent upon its location, its rate of generation, and the presence of reactive targets.

## Data Availability

The raw data supporting the conclusion of this article will be made available by the authors, without undue reservation.
