# Supplementary material for: Cell Origin and iNOS Function Are Critical to Macrophage Activation Following Acute Lung Injury
Source: Front Pharmacol. 2022 Jan 25;12:761496. doi: 10.3389/fphar.2021.761496 (PMC8822172; doi:10.3389/fphar.2021.761496)
Supplement: Supplementary file 1 [file Image1.pdf]

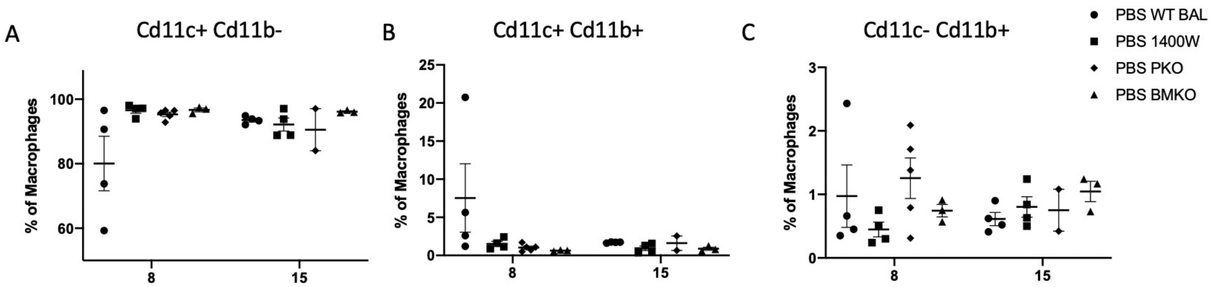

D

		8 Days								15 Days							
		Wild Type		1400W		PKO		BMKO		Wild Type		1400W		PKO		BMKO	
		Mean	SE	Mean	SE	Mean	SE	Mean	SE	Mean	SE	Mean	SE	Mean	SE	Mean	SE
Cd206	Cd11c+ Cd11b-	682	39.2	956	307.8	699	70.7	543	15.0	661	4.9	543	23.6	682	86.8	549	24.1
	Cd11c+ Cd11b+	1865	700.3	958	131.5	1135	162.9	624	8.3	1291	51.9	1126	116.2	1154	122.3	627	70.9
	Cd11c- Cd11b+	436	75.5	446	19.3	592	65.6	370	57.5	429	30.8	468	43.4	696	103.4	225	2.3
Ly6C	Cd11c+ Cd11b-	1060.5	37.9	1168.5	171.1	1226.4	118.8	1013.8	30.1	1361.3	23.5	1243.7	57.1	1471.8	267.4	1150.3	33.9
	Cd11c+ Cd11b+	1713.5	304.9	1598.0	186.7	1764.7	303.1	1115.3	66.3	1719.3	70.7	2365.9	270.1	3049.2	1037.0	1425.1	171.8
	Cd11c- Cd11b+	524.8	70.2	437.7	32.2	351.6	71.3	348.8	46.5	688.4	75.6	670.5	50.9	329.3	40.3	401.3	21.5

Supplementary Figure 1. BAL macrophage population maturity and phenotype are similar in PBS instilled controls. PBS instilled in wild type (circle), had similar proportion of mature (A), intermediate (B), and immature (C) macrophages as those also administered 1400W (square), PKO chimeras (diamond), and BMKO chimeras (triangle). Cd206 and Ly6C expression, as represented by MFI, remained constant across PBS instilled animals (D).
